# Optical Properties and Upconversion Luminescence of BaTiO_3_ Xerogel Structures Doped with Erbium and Ytterbium

**DOI:** 10.3390/gels8060347

**Published:** 2022-06-02

**Authors:** Ekaterina I. Lashkovskaya, Nikolai V. Gaponenko, Margarita V. Stepikhova, Artem N. Yablonskiy, Boris A. Andreev, Vadim D. Zhivulko, Alexander V. Mudryi, Igor L. Martynov, Alexander A. Chistyakov, Nikolai I. Kargin, Vladimir A. Labunov, Tamara F. Raichenok, Sergey A. Tikhomirov, Victor Yu. Timoshenko

**Affiliations:** 1 Laboratory of Nanophotonics, Belarusian State University of Informatics and Radioelectronics, P. Browki 6, 220013 Minsk, Belarus; katerinaxy90@gmail.com (E.I.L.); labunov@bsuir.by (V.A.L.); 2 Institute for Physics of Microstructures, Russian Academy of Sciences, GSP-105, 603950 Nizhny Novgorod, Russia; mst@ipmras.ru (M.V.S.); yablonsk@ipmras.ru (A.N.Y.); boris@ipmras.ru (B.A.A.); 3 Scientific-Practical Materials Research Centre of National Academy of Sciences of Belarus, 220072 Minsk, Belarus; zhivulko@ifttp.bas-net.by (V.D.Z.); mudryi@ifttp.bas-net.by (A.V.M.); 4 Institute for Nanoengineering in Electronics, Spintronics and Photonics, National Research Nuclear University “MEPhI”, 115409 Moscow, Russia; ILMartynov@mephi.ru (I.L.M.); AAChistyakov@mephi.ru (A.A.C.); NIKargin@mephi.ru (N.I.K.); 5 Stepanov Institute of Physics, National Academy of Sciences of Belarus, 220072 Minsk, Belarus; raitf@ifanbel.bas-net.by (T.F.R.); s.tikhomirov@ifanbel.bas-net.by (S.A.T.); 6 Faculty of Physics, Lomonosov Moscow State University, GSP-1, Leninskie Gory, 119991 Moscow, Russia; timoshen@physics.msu.ru

**Keywords:** barium titanate, erbium, ytterbium, upconversion, luminescence, sol-gel, xerogel, strontium titanate, microcavity

## Abstract

Erbium upconversion (UC) photoluminescence (PL) from sol-gel derived barium titanate (BaTiO_3_:Er) xerogel structures fabricated on silicon, glass or fused silica substrates has been studied. Under continuous-wave excitation at 980 nm and nanosecond pulsed excitation at 980 and 1540 nm, the fabricated structures demonstrate room temperature PL with several bands at 410, 523, 546, 658, 800 and 830 nm, corresponding to the ^2^H_9/2_ → ^4^I_15/2_, ^2^H_11/2_ → ^4^I_15/2_, ^4^S_3/2_ → ^4^I_15/2_, ^4^F_9/2_→ ^4^I_15/2_ and ^4^I_9/2_→ ^4^I_15/2_ transitions of Er^3+^ ions. The intensity of erbium UC PL increases when an additional macroporous layer of strontium titanate is used beneath the BaTiO_3_ xerogel layer. It is also enhanced in BaTiO_3_ xerogel films codoped with erbium and ytterbium (BaTiO_3_:(Er,Yb)). For the latter, a redistribution of the intensity of the PL bands is observed depending on the excitation conditions. A multilayer BaTiO_3_:(Er,Yb)/SiO_2_ microcavity structure was formed on a fused silica substrate with a cavity mode in the range of 650–680 nm corresponding to one of the UC PL bands of Er^3+^ ions. The obtained cavity structure annealed at 450 °C provides tuning of the cavity mode by 10 nm in the temperature range from 20 °C to 130 °C. Photonic application of BaTiO_3_ xerogel structures doped with lanthanides is discussed.

## 1. Introduction

Colour tuning and enhancement of anti-Stokes luminescence have been widely studied in various lanthanide-doped materials since the mid-1960s [1,2,3,4]. Low-cost coatings with upconversion photoluminescence (PL) of lanthanides are of interest for the rare side of solar cell panels as spectral converters [5,6]. The upconversion of erbium in barium titanate was recently studied [7,8,9]. The luminescence of trivalent erbium and other lanthanides is enhanced in microcavities and in the presence of sensitizing ions, in particular, ytterbium for erbium [2]. The luminescence enhancement of lanthanides in microcavities is associated with a redistribution of the optical mode density in the cavities. An enhancement of the Stokes luminescence and a narrowing of the erbium luminescence bands were observed in porous silicon [10,11] and in Si/SiO_2_ microcavities [12]. Europium luminescence and erbium upconversion luminescence was enhanced in sol-gel derived microcavities [13,14,15]. We recently reported that the erbium upconversion luminescence from xerogels can be enhanced when xerogels are fabricated on mesoporous and macroporous layers, such as porous strontium titanate xerogel [16]. The production of multilayer coatings increases the quality factor of the microcavity, but together with an increase in the required annealing temperature, it increases the cost of the product. From this point of view, it is of interest to develop various low-cost upconverter film technologies.

Another promising type of structures doped with lanthanides are tunable photonic crystals. Of practical interest is the development of photonic crystals with a photonic stop band corresponding to the emission band of the embedded species, so that when the photonic band gap shifts due to changes in external conditions, the luminescence characteristics of the embedded species should change accordingly. Developing the technology of synthesis of tunable photonic crystals corresponds to the challenge of inhibition or enhancement of the spontaneous emission and makes influence on laser physics, display technologies, light-emitting devices, and optical sensors. Several tunable photonic crystals, not yet doped with lanthanides, were made from artificial opals [17], filling their pores with VO_2_ [18] or BaTiO_3_ [19]. Materials such as VO_2_ and BaTiO_3_ exhibit a phase transition at the Curie temperature. As a result, a temperature induced shift of the photonic stop band was observed upon heating or cooling of the opal/VO_2_ and opal/BaTiO_3_ samples [18,19].

In this work, we study the optical properties of barium titanate xerogel films doped with lanthanides. The subject of investigation in the presented paper are multilayer structures from barium titanate xerogel codoped with erbium and ytterbium (BaTiO_3_:(Er,Yb)) exhibiting room temperature upconversion luminescence of Er^3+^ ions. In the first part of the paper, we characterize the upconversion luminescence from barium titanate multilayer xerogels (BaTiO_3_:(Er,Yb)) fabricated on a silicon wafer at a relatively high annealing temperature, namely 800 °C. The upconversion PL from BaTiO_3_:(Er,Yb) xerogels is compared with the case of BaTiO_3_:Er xerogels doped with erbium ions only, as well as with BaTiO_3_:(Er,Yb) xerogels, fabricated on a porous strontium titanate xerogel layer on silicon. In the second part, we describe BaTiO_3_:(Er,Yb)/SiO_2_ photonic crystals and microcavities. We show that upconversion PL is observed for a BaTiO_3_:(Er,Yb)/SiO_2_ microcavity with 7 layers of BaTiO_3_:(Er,Yb) xerogel after annealing of each layer at a relatively low temperature (450 °C), and this UC PL is enhanced after additional annealing of the structure at 600 °C. The microcavity structures exhibit a shift of the cavity mode: a blue shift from 650 to 680 nm in the transmission spectra caused by additional annealing of the structure at 600 °C and a shift of 10 nm (from 670 to 680 nm) in the reflectance spectra upon heating or cooling of the sample in the temperature range of 20–130 °C.

## 2. Experimental

Several sols were prepared to fabricate non-periodic and periodic multilayer structures with erbium-doped or erbium-ytterbium codoped barium titanate xerogel layers. The obtained samples (labeled as A, B, C, D, E and F) are shown in Table 1.

For the synthesis of barium titanate sol corresponding to BaTiO_3_:Er xerogel (sol I), titanium isopropoxide (Ti(OC_3_H_7_)_4_), barium acetate (Ba(CH_3_COO)_2_), erbium acetate hydrate (Er(CH_3_COO)_3_·xH_2_O), acetylacetone (CH_3_COCH_2_COCH_3_) and acetic acid (CH_3_COOH) were used as starting components. The amounts of titanium isopropoxide and barium acetate were chosen so that the Ti/Ba ratio corresponded to the stoichiometric composition of barium titanate in the films (i.e., Ti:Ba = 1:1). The mixture was stirred for 1 h with an electromechanical stirrer until all components were completely dissolved, resulting in the formation of a stable film-forming sol. Er(CH_3_COO)_3_·xH_2_O was added to the BaTiO_3_ sol to obtain BaTiO_3_ xerogel with 3 at.% of erbium.

The second sol (sol II) was prepared to fabricate a porous SrTiO_3_ sublayer [20]. A porous SrTiO_3_ film was deposited using a water containing sol which was synthesized as follows. First, titanium isopropoxide was dissolved in a mixture of ethylene glycol monomethyl ether and nitric acid to prevent gelation initiated by titanium isopropoxide. Then, strontium nitrate was dissolved in distilled water, followed by the addition of ethylene glycol monomethyl ether. Finally, both solutions were mixed to form a sol.

The third sol (sol III) was prepared for the synthesis of BaTiO_3_:(Er,Yb) xerogel, namely Ba_0.76_Er_0.04_Yb_0.20_TiO_3_, according to the following procedure. Two solutions were prepared. Titanium isopropoxide (Ti(OC_3_H_7_)_4_) was dissolved in acetylacetone (CH_3_COCH_2_COCH_3_), stirred until the solution cooled. Barium acetate (Ba(CH_3_COO)_2_) was separately dissolved in distilled water and stirred until complete dissolution. Erbium acetate hydrate (Er(CH_3_COO)_3_·xH_2_O) was added to the barium acetate solution and stirred until complete dissolution. Then, ytterbium acetate hydrate (Yb(CH_3_COO)_3_·xH_2_O) was added to the solution of barium and erbium acetates and stirred until complete dissolution. Acetic acid (CH_3_COOH) was added to the solution of Ba, Er, and Yb acetates and stirred for about 5 min. Finally, the solutions of titanium isopropoxide and Ba, Er and Yb acetates were mixed and stirred for about 5 min. Ethanol (C_2_H_5_OH) was then added to this solution and stirred for 1.5 h.

To obtain silica xerogel (SiO_2_), silica sol (sol IV) was prepared. Concentrated nitric acid (HNO_3_) was added to an alcohol-water mixture (volume ratio of distilled water and ethanol (C_2_H_5_OH) approximately 1:6) to pH = 1. Tetraethyl orthosilicate (Si(OC_2_H_5_)_4_) was added to this mixture, stirred and the pH was adjusted again to 1 by adding concentrated nitric acid. The final solution was stirred for half an hour. The sol should age for at least 24 h in airtight conditions before the deposition.

To form a porous SrTiO_3_ xerogel layer, sol II was deposited by spinning at the rate of 2700 rpm for 30 s on a monocrystalline silicon wafer. Each deposited layer was dried at 200 °C for 10 min, then the next layer was deposited and dried in the same way. The deposition was repeated seven times in order to obtain a film of the required thickness. The final calcination was carried out at 800 °C for 40 min in air.

Samples A and B with BaTiO_3_:Er xerogel layers were fabricated by depositing sol I on a Si substrate and on a porous-SrTiO_3_/Si substrate, respectively. After the deposition of sol I by spinning directly on a Si substrate (sample A) or on a preliminary deposited porous SrTiO_3_ xerogel layer (sample B), it was dried at 200 °C for 10 min and annealed at 450 °C for 30 min. Nine layers were prepared in this way. The samples were then finally heat treated at 800 °C for 1 h in air.

Samples C and D with BaTiO_3_:(Er,Yb) xerogel layers on a Si substrate and on a porous-SrTiO_3_/Si substrate, respectively, were fabricated from the sol III under the same deposition and annealing conditions as samples A and B.

Sample E (Bragg reflector) containing 3 pairs of alternating BaTiO_3_:(Er,Yb) and SiO_2_ layers was fabricated by depositing sols III and IV on a glass substrate. Each layer was dried at 200 °C for 10 min and annealed at 450 °C for 30 min.

Finally, sample F (BaTiO_3_:(Er,Yb)/SiO_2_ microcavity) was fabricated by depositing sols III and IV on a fused silica substrate under the same conditions as for the sample E. The microcavity structure included: a lower Bragg reflector, consisting of three pairs of BaTiO_3_:(Er,Yb) and SiO_2_ layers, a thicker BaTiO_3_:(Er,Yb) xerogel layer, and an upper Bragg reflector, also consisting of three SiO_2_/BaTiO_3_:(Er,Yb) pairs. This structure was additionally annealed at 500 °C and 600 °C for 30 min.

The morphology of the obtained samples was examined using an S-4800 scanning electron microscope (SEM) (Hitachi, Japan) equipped with Quntax 200 Bruker for energy dispersive X-ray analyses (EDX). Platinum was sputtered onto the samples E and F fabricated on the glass and fused silica substrates.

To estimate the content of Er and Yb in the obtained xerogels, xerogel powders were derived from the sol I (BaTiO_3_:Er) and sol III (BaTiO_3_:(Er,Yb)) by heat treatment at 1000 °C. The prepared xerogel powders were analyzed by EDX analyses.

The depth distribution of elements in the obtained samples and their 3D images were studied using secondary-ion mass spectrometry (SIMS) (TOF.SIMS 5, IONTOF, Munster, Germany). Secondary positive ions were obtained by bombarding the samples with Bi^+^ ions with an energy of 30 keV (1 pA). Samples were etched in situ by sputtering with a focused (150 µm^2^) beam of Cs^+^ ion with an energy of 2 keV (100 nA) by raster scanning over the sample surface.

Studies of upconversion photoluminescence were carried-out in the regimes of continuous or pulsed optical excitation. A focused 980 nm laser beam of a 200 mW diode module was used for the excitation of upconversion PL (power density J ~ 10 W/cm^2^) in the continuous-wave (CW) mode. The emission in the visible range was focused on the entrance slit of a 0.6 m grating spectrometer equipped with 1200 gr/mm gratings, and the PL intensity was measured using an R 9110 Hamamatsu photomultiplier tube (PMT) sensitive in the spectral range of 200–850 nm. The spectral resolution of the PL measurement system was ~0.6 nm. A lock-in-amplifier with mechanical chopping at a frequency of about 20 Hz was used for signal recovery.

Time-resolved upconversion PL spectra were measured using an optical parametric oscillator (OPO) pumped with the third harmonic (355 nm) of a pulsed Nd:YAG laser and tuned in the spectral range from 700 nm to 1600 nm. The OPO pulse duration was 10 ns, the repetition rate was 10 Hz, and the average power was ~5 mW. Registration of up-conversion PL in the visible range was carried out using an Acton 2300i grating spectrometer, a Si-based PMT (350–800 nm), and a LeCroy digital oscilloscope.

The reflection and transmission spectra were measured on a Cary-500 ScanUV-VIS-NIR spectrophotometer (Varian USA–Australia) and an MS122 spectrophotometer (PROSCAN Special Instruments, Belarus).

Reflection spectra studied in the temperature range of 20–130 °C were measured using an Ocean Optics USB2000+ spectrometer equipped with a fiber reflection probe and a halogen lamp. During the measurements, the sample was placed horizontally on the heating table, and the sample surface temperature was monitored by a thermocouple.

X-ray diffraction (XRD) studies of the films were carried out with a DRON-3 diffractometer using monochromatic Cu K*α* radiation with an exposition of 2 s per 0.04*°* step at room temperature.

## 3. Results and Discussion. Part I. Upconversion PL from Multilayer BaTiO_3_:(Er,Yb) Xerogel Structures on Porous SrTiO_3_/Si

**Figure 1:**
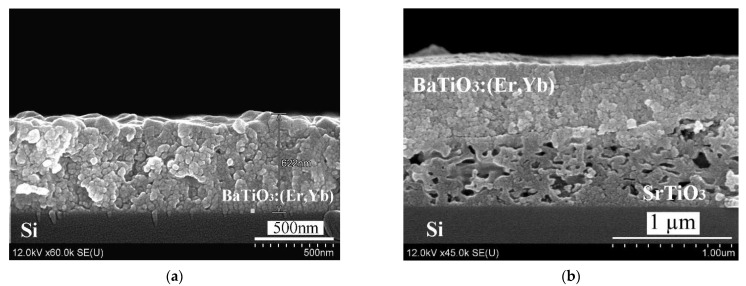
SEM-images of BaTiO_3_:(Er,Yb) xerogel layer deposited directly on a monocrystalline Si wafer (Sample C) (**a**) and on the same wafer using an intermediate macroporous SrTiO_3_ xerogel sublayer (Sample D) (**b**).

Figure 1 shows SEM-images of BaTiO_3_:(Er,Yb) xerogel layers deposited on a monocrystalline silicon wafer with and without an intermediate porous SrTiO_3_ sublayer. The deposition of BaTiO_3_:(Er,Yb) xerogel layer was performed in several steps. At each step, a relatively thin (~ 80 nm) BaTiO_3_:(Er,Yb) layer was fabricated by spinning and annealing. The total thickness of the resulting BaTiO_3_:(Er,Yb) xerogel layer was about 620 nm. The porous SrTiO_3_ sublayer had approximately the same thickness (~600 nm), so the entire thickness of the BaTiO_3_:(Er,Yb)/SrTiO_3_ structure was about 1.2 μm (Figure 1b). The morphology of samples A and B was similar to that of samples C and D, respectively [16].

According to EDX studies of the xerogel powders, in the powder sample prepared from the sol I (doped with erbium only) the average concentration of Er was 3.8 at.% with the standard deviation of 0.8 at.%, while in the powder sample prepared from the sol III (with Er and Yb) the average concentration of Er and Yb was 1.1 and 5.4 at.%, respectively, with the standard deviation of 0.6 and 0.1 at.%.

**Figure 2:**
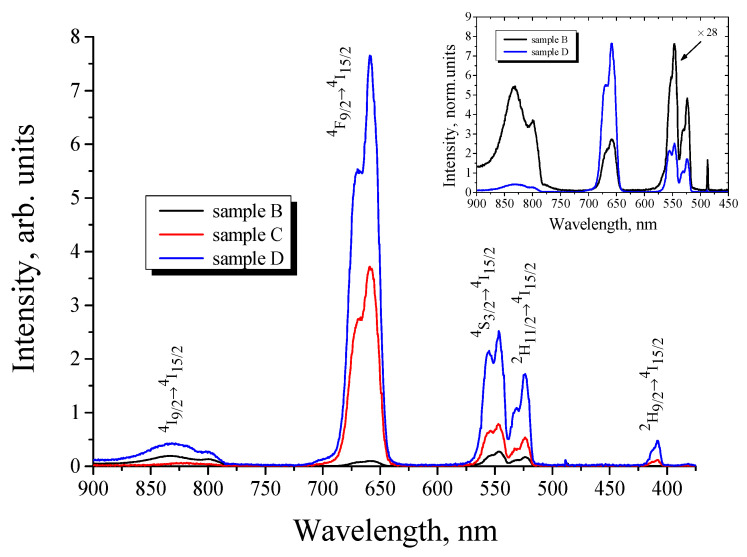
Upconversion PL spectra of BaTiO_3_:Er (sample B) and BaTiO_3_:(Er,Yb) (samples C and D) xerogel film structures on a Si substrate, taken under CW excitation at the wavelength of 980 nm. Samples B and D were obtained using an additional macroporous SrTiO_3_ xerogel sublayer before the deposition of the active BaTiO_3_ layer. Inset: comparison of the PL spectra of the samples B (multiplied by 28) and D.

The comparison of BaTiO_3_:Er xerogel film structures deposited directly on a monocrystalline Si substrate (sample A) and obtained using an additional macroporous SrTiO_3_ xerogel sublayer between the Si substrate and BaTiO_3_:Er (sample B) showed a significant increase of the upconversion PL intensity in the sample with the SrTiO_3_ sublayer. The upconversion PL spectrum of the sample B taken under CW excitation at a wavelength of 980 nm is shown in Figure 2. For the sample A without a porous SrTiO_3_ sublayer, upconversion PL was not detectable under the same excitation conditions [16]. The reason for the enhancement of the upconversion luminescence of erbium-doped xerogel could be the multiple scattering of the excited light in the presence of porous strontium titanate layer [16]. The observed upconversion PL spectra are characterized with several PL bands at 410, 523, 546, 658, 800 and 830 nm corresponding to ^2^H_9/2_ → ^4^I_15/2_, ^2^H_11/2_ → ^4^I_15/2_, ^4^S_3/2_ → ^4^I_15/2_, ^4^F_9/2_→ ^4^I_15/2_ and ^4^I_9/2_→ ^4^I_15/2_ transitions of trivalent erbium ions. A significant enhancement of upconversion PL was observed for BaTiO_3_ xerogel films codoped with Er and Yb ions (samples C and D). As can be seen in Figure 2, an intensive upconversion PL under CW excitation at 980 nm was observed for BaTiO_3_:(Er,Yb) xerogel films obtained both with (sample D) and without (sample C) a porous SrTiO_3_ sublayer. For both samples the overall intensity of upconversion PL was more than an order of magnitude higher in comparison with the BaTiO_3_:Er layer doped only with Er ions.

It can also be seen in Figure 2 that codoping with Yb ions leads to a significant redistribution of the PL intensity between the bands in the green (550 nm) and red (660 nm) spectral ranges. The inset in Figure 2 shows a comparison of the PL spectra of BaTiO_3_:(Er,Yb) and BaTiO_3_:Er xerogel films deposited on the SrTiO_3_/Si substrates. For the BaTiO_3_:Er film the most intensive band (546 nm) corresponds to the ^4^S_3/2_ → ^4^I_15/2_ transition of Er^3+^ ions, while for the BaTiO_3_:(Er,Yb) sample the PL spectrum is dominated by the red line (658 nm) corresponding to the ^4^F_9/2_→ ^4^I_15/2_ transition. Such redistribution of the intensity between the green and red upconversion PL bands with an increase in Yb content is well known for different types of upconversion materials co-doped with Er and Yb [21,22,23,24] and is attributed to the cross-relaxation between adjacent Yb^3+^ and Er^3+^ ions and the multiphonon relaxation in Er^3+^ ions.

**Figure 3:**
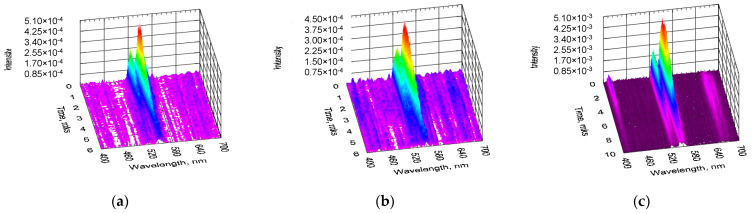
Time-resolved spectra of upconversion PL obtained from BaTiO_3_:Er (sample B) (**a**,**b**) and BaTiO_3_:(Er,Yb) (sample D) (**c**) xerogel layers deposited on a silicon wafer using a porous SrTiO_3_ xerogel sublayer. The spectra were measured under pulsed excitation with the wavelengths of λ_ex_ = 980 nm (**a**,**c**) and λ_ex_ = 1540 nm (**b**). The intensity of the upconversion PL signal is plotted as a function of the wavelength and the time delay after the laser pulse.

Figure 3 shows time-resolved spectra of upconversion PL obtained under pulsed optical excitation from BaTiO_3_:Er and BaTiO_3_:(Er,Yb) xerogel layers deposited on the SrTiO_3_/Si substrates. For the BaTiO_3_:Er/SrTiO_3_/Si structure (Sample B) upconversion PL was detected under excitation both at 980 nm (Figure 3a) and 1540 nm (Figure 3b), while for the BaTiO_3_:(Er,Yb)/SrTiO_3_/Si structure (Sample D) intensive upconversion PL was observed primarily for the excitation wavelengths around 980 nm (Figure 3c).

The latter is apparently due to the dominating contribution of Yb ions into the absorption of IR light in the BaTiO_3_ layers codoped with Er and Yb [21,22,23,24]. It should be noted that for all the samples under study, upconversion PL spectra obtained under pulsed optical pumping, regardless of the excitation wavelength, were dominated by the green emission line around 550 nm corresponding to the ^2^H_11/2_ → ^4^I_15/2_ and ^4^S_3/2_ → ^4^I_15/2_ radiative transitions. This can be associated with a significantly higher excitation power density under pulsed pumping conditions. It is well known [23,25] that an increase in the excitation power density leads to a redistribution of the intensities between the red and green upconversion luminescence bands of Er^3+^ ions corresponding to the ^4^S_3/2_ → ^4^I_15/2_ and ^4^F_9/2_ → ^4^I_15/2_ transitions, respectively.

As in the case of CW pumping, under pulsed optical excitation, the PL intensity of the Yb codoped samples was about an order of magnitude higher in comparison with the similar BaTiO_3_ xerogel layers doped only with Er ions (Figure 3a,c). For the BaTiO_3_:(Er,Yb) xerogel layers, less intensive upconversiton PL bands at ~410 nm and ~660 nm corresponding to the ^2^H_9/2_ → ^4^I_15/2_ and ^4^F_9/2_ → ^4^I_15/2_ transitions of Er^3+^ ions were also observed in the time-resolved PL spectra (Figure 3c).

**Figure 4:**
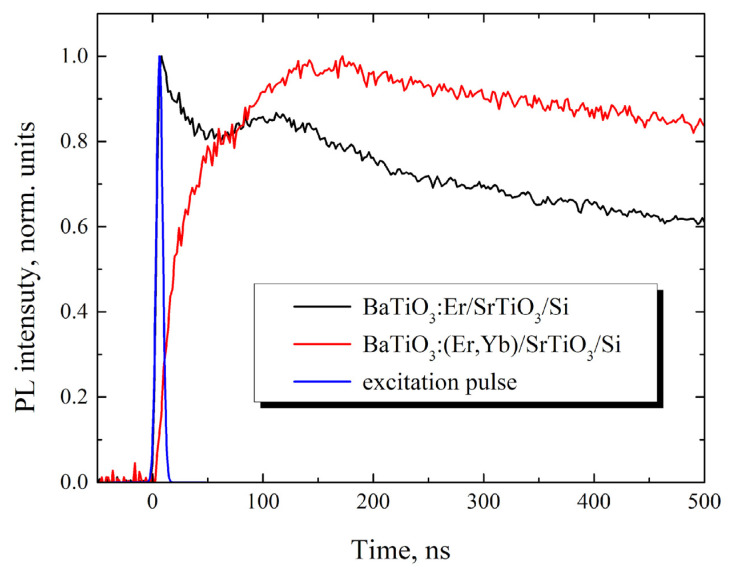
Time dependencies of the upconversion PL intensity (at 550 nm) observed in the BaTiO_3_:Er (sample B) and BaTiO_3_:(Er,Yb) (sample D) xerogel layers deposited on the SrTiO_3_/Si substrate. The PL kinetics were obtained under pulsed excitation at 980 nm with the pulse duration of 10 ns (blue curve). Each curve was normalized to the maximum value of the PL intensity.

To find out the excitation mechanism of upconversion PL in the obtained structures, we have compared time dependences of the PL intensity for the main upconversion PL band (at 550 nm) of the BaTiO_3_:Er (sample B) and BaTiO_3_:(Er,Yb) (sample D) xerogel layers deposited on the SrTiO_3_/Si substrate (Figure 4). For the BaTiO_3_ layers doped only with Er^3+^ ions we have observed two components in the growth dynamics of the PL intensity with the characteristic growth times of about 2 ns and 30–40 ns (black curve in Figure 4).

**Figure 5:**
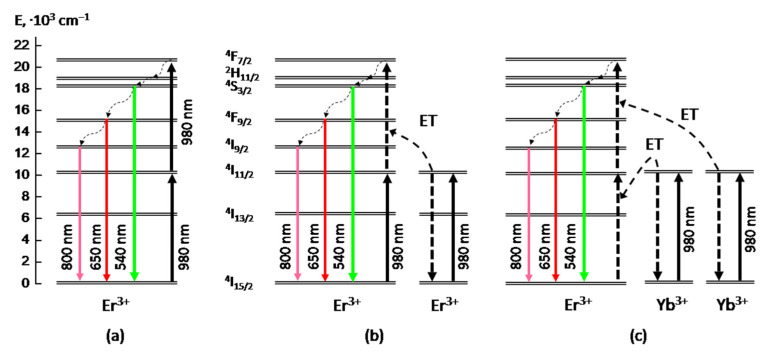
Energy level diagram showing mechanisms of upconversion PL excitation under 980 nm optical pumping: (**a**) “excited state absorption” (ESA) and (**b**) “energy transfer upconversion” (ETU) in Er-doped material; (**c**) “energy transfer upconversion” in Er/Yb-codoped material. “ET”—energy transfer.

We assume that these two components correspond to the two different excitation mechanisms of upconversion PL of Er^3+^ ions in the obtained BaTiO_3_:Er layers, namely “excited state absorption” (ESA) and “energy transfer upconversion” (ETU) [5,26,27], which both act under these excitation conditions. The first component (faster) of the upconversion PL apparently occurs as a result of the almost simultaneous absorption of two photons at the ^4^I_15/2_ → ^4^I_11/2_ and ^4^I_11/2_ → ^4^F_7/2_ transitions and subsequent nonradiative relaxation to the ^4^S_3/2_ level (ESA mechanism, Figure 5a). So the faster growth time value (~2 ns) evidently corresponds to the front edge of the excitaition laser pulse (blue curve in Figure 4). The second (slower) stage of the upconversion PL growth with the characteristic time of 30–40 ns can be associated with the energy transfer between two excited Er^3+^ ions in the framework of the so-called “energy transfer upconversion” (ETU) mechanism (Figure 5b).

In contrast to BaTiO_3_:Er samples, in BaTiO_3_:(Er,Yb) xerogel layers codoped with Er and Yb ions, we have observed only slow component in the upconversion PL growth with the characteristic time of about 40 ns (red curve in Figure 4), which is close to the corresponding value obtained for the BaTiO_3_:Er layers. This result means that in the obtained BaTiO_3_:(Er,Yb) xerogel layers the dominant mechanism of upconversion of IR radiation near 980 nm is the absorption by Yb ions and the energy transfer from Yb to Er ions (Figure 5c). In this case, the characteristic rise time (~40 ns) of the main upconversion PL band at 550 nm is apparently determined by the time of energy transfer from Yb to Er ions.

Returning to the posible reasons for the influence of the SrTiO_3_ layer on the efficiency of upconversion PL, we assume that the presence of such a porous layer coated with relatively dense barium titanate doped with erbium and ytterbium provides the presence of erbium and ytterbium in the meso- and macropores of the strontium titanate layer and on the interface between the porous strontium titanate layer and the denser barium titanate layer [16]. Thus, an erbium containing inhomogeneous structure is formed, which consists of components with low and high refractive indices, in particular, air in the volume of the pores and islands of perovskites. The resulting inhomogeneous medium can scatter the excitation light, especially taking into account the high refractive index *n* of strontium titanate (*n* ≈ 2 for strontium titanate films depending on the fabrication technology and *n* > 2 for the crystal). Enhanced scattering of the excitation light leads to its effective absorption coefficient and absorption cross-section of ytterbium and erbium in porous perovskite media. Excitation light scattering in inhomogeneous porous media can play an important role in the enhancement of luminescence and random lasing. The effect of light scattering on the luminescence of porous materials, as well as of dyes and lanthanides embedded in porous matrices has been previously reported in the works concerning porous Si nanowires [28,29], embedded dyes [30,31] and Eu- and Tb-doped xerogels embedded in porous anodic alumina [32,33,34].

## 4. Results and Discussion. Part II. Upconversion in Microcavity

**Figure 6:**
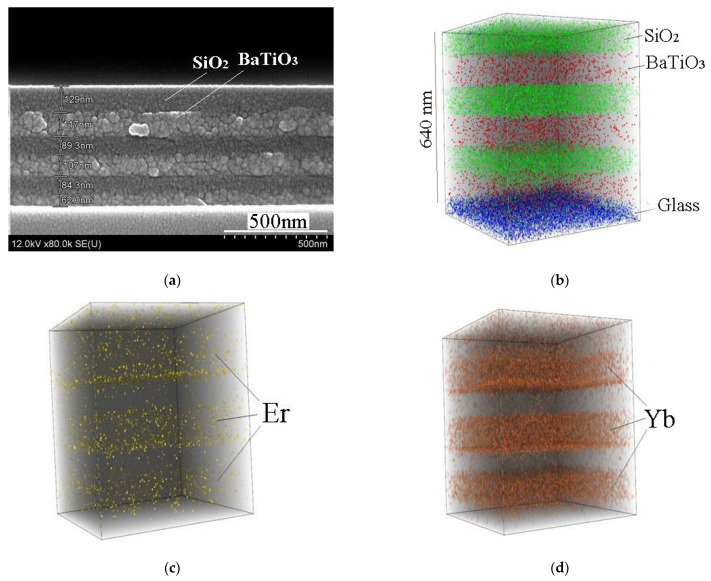
SEM image (**a**) and 3D SIMS images (**b**–**d**) of the BaTiO_3_:(Er,Yb)/SiO_2_ Bragg reflector on a glass substrate (sample E) after annealing at 450 °C for 30 min in air.

Figure 6 shows SEM and 3D SIMS images of sol-gel derived BaTiO_3_:(Er,Yb)/SiO_2_ Bragg reflector fabricated on a glass substrate (sample E). Each BaTiO_3_ and SiO_2_ layer was dried after spin-on deposition and then annealed at 450 °C for 30 min. From the SIMS data, we may see that each BaTiO_3_ layer is enriched with Er and Yb, and Yb concentration dominates. Penetration of Er and Yb in SiO_2_ is negligible. Noteworthy, the total thickness of BaTiO_3_:Er:Yb layers is about 300 nm and under continuous wave excitation at the wavelength of 980 nm the erbium upconversion luminescence from this sample annealed at 450 °C was not observed.

**Figure 7:**
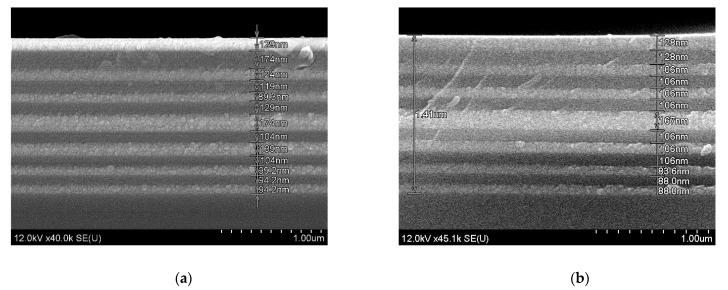
SEM images of the BaTiO_3_/SiO_2_ microcavity (sample F) after heat treatment at 450 °C (**a**) and 600 °C (**b**).

Further, the same conditions of sols deposition and annealing of the xerogel layers were applied to fabricate a microcavity structure on a fused silica substrate (sample F). The total thickness of the microcavity was about 1.5 µm after heat treatment at 450 °C (Figure 7a) and about 1.4 µm after additional annealing at 600 °C (Figure 7b). We have to note, that contrary to thick BaTiO_3_ xerogel films annealed at 800 °C [16] and xerogel powder annealed at 1000 °C, thin BaTiO_3_ xerogel films of microcavity structures annealed at 450 °C and 600 °C are X-ray amorphous.

**Figure 8:**
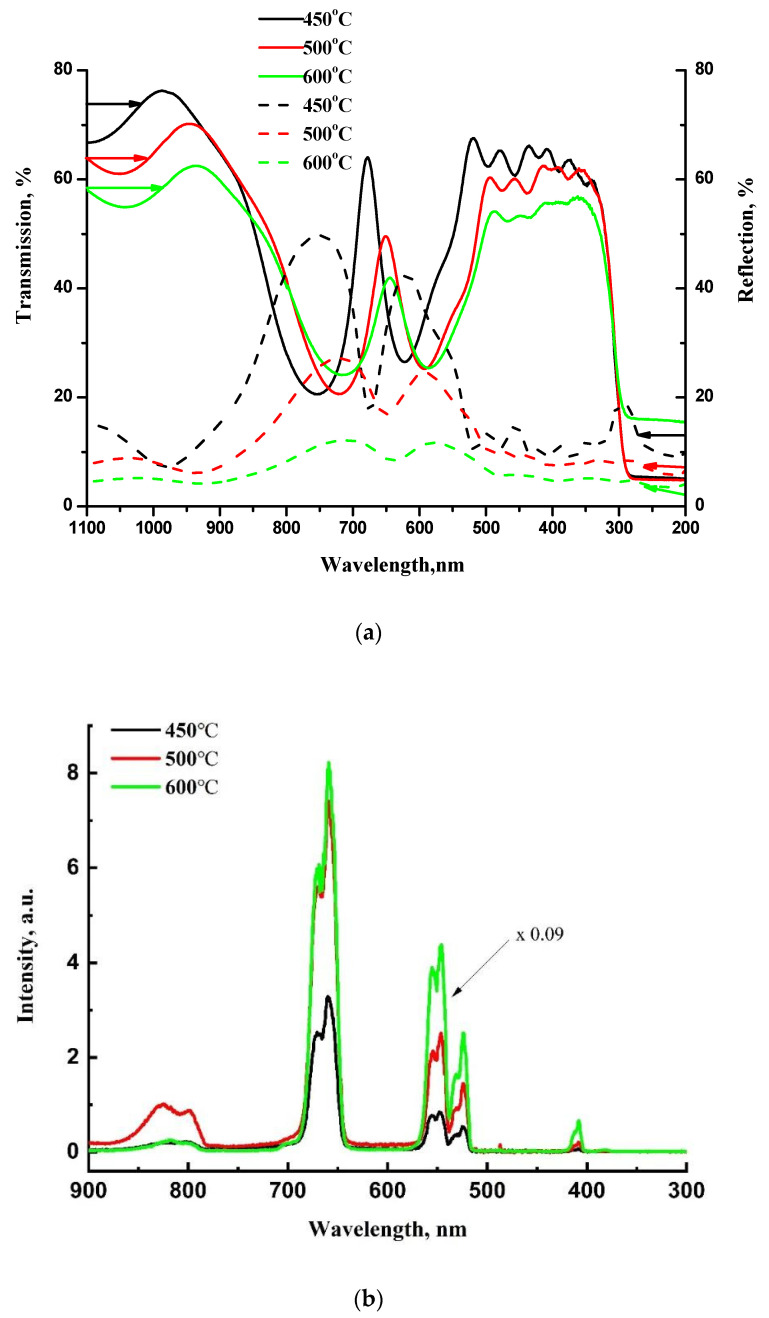
Room-temperature optical spectra of BaTiO_3_:(Er,Yb)/SiO_2_ microcavity (sample F): (**a**) transmission (solid lines) and reflection (dashed lines) spectra of BaTiO_3_/SiO_2_ microcavity on a fused silica after final annealing at 450 °C (black lines), 500 °C (red lines) and 600 °C (green lines); (**b**) upconversion PL spectra of the microcavity obtained after final annealing at 450 °C (black line), 500 °C (red line) and 600 °C (green line). Each barium titanate layer is doped with Er and Yb.

In contrast to the above-mentioned single Bragg reflector, the microcavity demonstrates erbium upconversion luminescence after similar thermal annealing of each layer at 450 °C for 30 min in air (Figure 8b). Note that such an upconverter is inexpensive, can be formed on a low-cost substrate, such as glass, and does not require vacuum or special gases for deposition and annealing. The transmission and reflection spectra shown in Figure 8a confirm the formation of a microcavity with a cavity mode at 680 nm after annealing at 450 °C. As we reported earlier, additional heat treatment at elevated annealing temperatures results in a blue shift of the sol-gel derived BaTiO_3_/SiO_2_ photonic band gap [35] and cavity mode [36]. For our microcavity structure, we also observed a blue shift of the cavity mode after additional annealing of the structure at 500 °C and 600 °C (Figure 8a), and the transparency of the structure decreased over the entire measurement range, probably due to thermally induced diffuse scattering.

The intensity of Er upconversion PL increases with the annealing temperature, and for the sample annealed at 600 °C it is about 30 times higher as compared with the sample annealed at 450 °C. Despite the shift of the cavity mode and the change in transparency due to an increase of the annealing temperature, no significant intensity redistribution between the luminescence bands is observed. This result can be understood by taking into account the low-quality factor Q of the microcavity with Bragg reflectors consisting of only 3 BaTiO_3_/SiO_2_ pairs bordering the half-wave layer. The Q factors estimated as λ/Δλ (the ratio of the resonant wavelength to the full width at half maximum of the resonant peak) amount to 14, 13, and 11 for the microcavity structure annealed at 450 °C, 500 °C, and 600 °C, respectively. The data were obtained from the transmission spectra using the Lorentz fit of the spectra.

The shift in the cavity mode is the result of the change in the refractive index *n* multiplied by the film thickness *d* (optical path length, *nd*) of the SiO_2_ and BaTiO_3_ films. We observe a blue shift of the cavity mode for the microcavity annealed at 450 °C or 600 °C. This effect is predictable considering that the value of *nd* decreases for both SiO_2_ and BaTiO_3_ with increasing annealing temperature. It is known that after high temperature annealing, xerogel film densification occurs, so *d* decreases by 10–30% and *n* increases by about 3–5% [37,38,39]. The same shrinkage effect was revealed for our samples. For example, after annealing at 600 °C, the film thickness decreases by about 12% and 8% for SiO_2_ and BaTiO_3_ films, respectively (Figure 7). Thus, a net decrease of *nd* occurs after the heat treatment, and the higher is annealing temperature, the stronger blue shift of the cavity mode occurs.

With an increase in the annealing temperature, the transmission at an excitation wavelength of 980 nm decreases: T = 76%, 67% and 59% for the structures annealed at 450 °C, 500 °C and 600 °C, respectively (Figure 8a). Reflection also decreases at higher annealing temperatures and amounts to 8%, 7% and 5% for the annealing temperatures of 450 °C, 500 °C and 600 °C, respectively. Thus the sum of absorption A and scattering S (A + S = 1 − T − R) increases with an increase in the annealing temperature, which may be the reason for the enhancement of luminescence at higher annealing temperatures.

**Figure 9:**
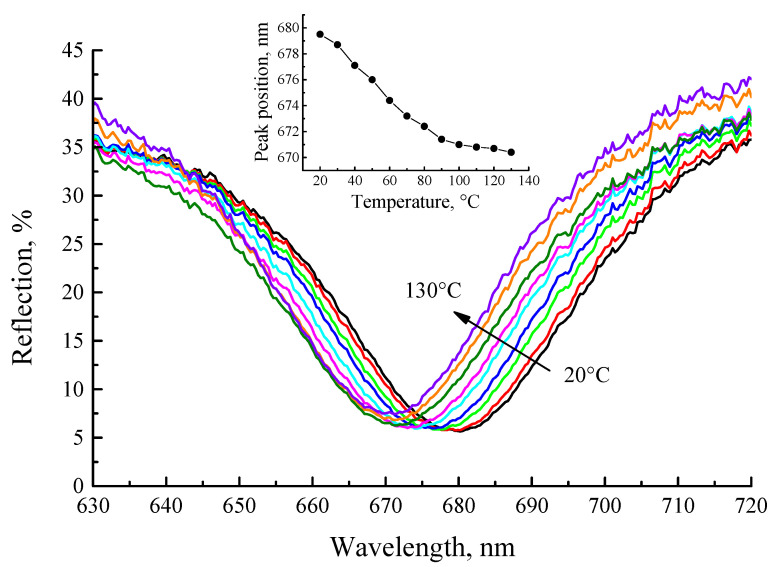
Fragments of the reflection spectra of the BaTiO_3_:(Er,Yb)/SiO_2_ microcavity (sample F, annealed at 450 °C) in the vicinity of the cavity mode, measured in the temperature range 20–130 °C. Inset: central wavelength of the cavity mode as a function of temperature.

Finally, we studied the shift of the cavity mode with detection temperature, as we observed earlier [35]. Figure 9 shows the reflection spectra of the cavity after annealing at 450 °C. A 10 nm blue shift of the cavity mode (from 680 nm to 670 nm) is observed upon heating of the cavity structure from 20 °C to 130 °C. It should also be noted that the reflectance of the microcavity structure increases upon sample heating, and a change of the reflection within 10% for the spectral ranges ~ 610 and ~ 750 nm is observed.

The luminescent properties of light emitting structures depend on the density of optical modes [40]. In microcavities and photonic crystals, the luminescence spectrum, the spontaneous emission rate and the luminescence indicatrix correlate with the transmission and reflection spectra. For this reason, a tunable light-emitting microcavity whose properties are sensitive to the external conditions such as temperature, pressure, field strength, could be an optical environment sensor. Considering that the luminescence of lanthanides in optically anisotropic structures depends on the photon density of states [41], in particular, the PL band width [10], PL intensity [11,12,13,14,15,29] and spontaneous emission rate [12] depend on the shift of the cavity mode, our further research will be aimed at improving the quality factor of the sol-gel derived BaTiO_3_/SiO_2_ microcavities doped with lanthanides and their application for optical monitoring [42,43].

## 5. Conclusions

In conclusion, strong upconversion luminescence of trivalent erbium ions was observed at room temperature from sol-gel derived Ba_0.76_Er_0.04_Yb_0.20_TiO_3_ xerogel structures under continuous-wave excitation at 980 nm and nanosecond pulsed excitation at 980 and 1540 nm. The upconversion luminescence spectra contain several bands at 410, 523, 546, 658, 800 and 830 nm, which correspond to the ^2^H_9/2_ → ^4^I_15/2_, ^2^H_11/2_ → ^4^I_15/2_, ^4^S_3/2_ → ^4^I_15/2_, ^4^F_9/2_→ ^4^I_15/2_ and ^4^I_9/2_→ ^4^I_15/2_ transitions in trivalent erbium ions. The intensity of upconversion emission in BaTiO_3_:(Er,Yb) structures co-doped with Er and Yb ions is more than an order of magnitude higher than in BaTiO_3_:Er structures doped only with erbium. It can be further increased by using an additional macroporous SrTiO_3_ sublayer beneath the BaTiO_3_ xerogel layer. A multilayer BaTiO_3_:(Er,Yb)/SiO_2_ microcavity structure was formed on a fused silica substrate with a cavity mode in the wavelength range of 650–680 nm corresponding to one of the upconversion luminescence bands. The spectral position of the cavity mode depends on the annealing temperature, as well as on the sample temperature during the measurements. Taking into account that the resulting microcavity structure exhibits intense upconversion luminescence at room temperature (after annealing at 600 °C), as well as a noticeable shift of the resonance mode (by 10 nm) upon heating of the structure from 20 °C to 130 °C, we consider such structures to be promising for optical monitoring, thus the upconversion luminescence variation upon the temperature in such structures will be the subject of our future studies.

## Figures and Tables

**Table 1:** Description of the samples under study with BaTiO_3_:Er and BaTiO_3_:(Er,Yb) xerogel films.

Sample Label	Type of Sol	Type of Xerogel	Substrate and Sublayer
Part I			
Sample A	Sol I	BaTiO_3_:Er	Si
Sample B	Sol I, Sol II	BaTiO_3_:Er	porous-SrTiO_3_/Si
Sample C	Sol III	BaTiO_3_:(Er,Yb)	Si
Sample D	Sol III, Sol II	BaTiO_3_:(Er,Yb)	porous-SrTiO_3_/Si
Part II			
Sample E	Sol III, Sol IV	Bragg reflector with 3 pairs of alternating layers BaTiO_3_:(Er,Yb)/SiO_2_	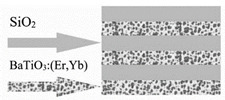 Glass
Sample F	Sol III, Sol IV	Microcavity BaTiO_3_:(Er,Yb)/SiO_2_	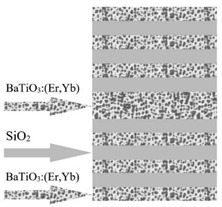 Fused silica

## Data Availability

Not applicable.
